# Antibacterial and Antiviral Roles of a Fish β-Defensin Expressed Both in Pituitary and Testis

**DOI:** 10.1371/journal.pone.0012883

**Published:** 2010-12-20

**Authors:** Jun-Yan Jin, Li Zhou, Yang Wang, Zhi Li, Jiu-Gang Zhao, Qi-Ya Zhang, Jian-Fang Gui

**Affiliations:** State Key Laboratory of Freshwater Ecology and Biotechnology, Wuhan Center for Developmental Biology, Institute of Hydrobiology, Graduate School of the Chinese Academy of Sciences, Chinese Academy of Sciences, Wuhan, China; New York University, United States of America

## Abstract

Defensins are a group of cationic peptides that exhibit broad-spectrum antimicrobial activity. In this study, we cloned and characterized a β-defensin from pituitary cDNA library of a protogynous hermaphroditic orange-spotted grouper (*Epinephelus coioides*). Interestingly, the β-defensin was shown to be dominantly expressed in pituitary and testis by RT-PCR and Western blot analysis, and its transcript level is significantly upregulated in reproduction organs from intersexual gonad to testis during the natural and artificial sex reversal. Promoter sequence and the responsible activity region analyses revealed the pituitary-specific POU1F1a transcription binding site and testis-specific SRY responsible site, and demonstrated that the pituitary-specific POU1F1a transcription binding site that locates between −180 and −208 bp is the major responsible region of grouper β-defensin promoter activity. Immunofluorescence localization observed its pituicyte expression in pituitary and spermatogonic cell expression in testis. Moreover, both *in vitro* antibacterial activity assay of the recombinant β-defensin and *in vivo* embryo microinjection of the β-defensin mRNA were shown to be effective in killing Gram-negative bacteria. And, its antiviral role was also demonstrated in EPC cells transfected with the β-defensin construct. Additionally, the antibacterial activity was sensitive to concentrations of Na^+^, K^+^, Ca^2+^ and Mg^2+^. The above intriguing findings strongly suggest that the fish β-defensin might play significant roles in both innate immunity defense and reproduction endocrine regulation.

## Introduction

Innate immunity is the first defense line against invading pathogens [1]. Defensins, as 3- to 6-kDa cationic peptides, are important effect molecules. They can rapidly kill microorganism pathogens including bacteria [2], mycobacteria, fungi [3], and viruses [4]. Based on their cysteine disulphide bonding, defensins are classified into α-, β- and θ-defensins [5]. α- and β-defensins distribute broadly in plants [6], [7], invertebrate [8] and vertebrate animals [9], but θ-defensins are only expressed in rhesus macaque [10]. More than 30 defensins in human and mouse [11] and 14 β–defensins in birds [12], [13], [14] were identified, but only 3, 2 and 2 β-defensin genes were respectively found in zebrafish, Fugu and Tetraodon by using a database mining approach. In zebrafish, constitutive expression of 3 β-defensins had been analyzed in healthy adult tissues [15]. In rainbow trout, β-defensin-1 was proved to have antiviral activity [16], and β-defensin-2, β-defensin-3 and β-defensin-4 were inducible with polyI:C treatment [17]. In olive flounder, the multiple β-defensins could be induced by pathogenic exposure [18]. Recently, we cloned a novel medaka β-defensin, and revealed its antimicrobial activity-specific to Gram-negative bacteria. Moreover, its immune modulation was demonstrated to be mediated by NF-κB and Sp1 [19].

β-defensins have been revealed to have more other biological activities in addition to their broad-spectrum antimicrobial roles [20], [21], [22], [23], [24]. For example, some β-defensins are chemoattractants for monocytes, lymphocytes and dendritic cells, which act as a link between innate and adaptive immune responses [25], [26], [27]. In *Torenia fournieri*, a unique plant with a protruding embryo sac, the defensin-like polypeptide LUTEs are pollen tube attractants secreted form synergid cells [28]. And, some testis- or epididymis-specific β-defensins, such as Bin1b [29] and EP2 [30], [31], [32], have been identified in mammals. These findings suggest that some β-defensins might be related to reproduction endocrine regulation.

Several β-defensins have been identified in brain or testis [29], [33], [34], but both pituitary and testis-produced β-defensins have not been reported. In this study, we cloned and characterized a β-defensin from pituitary cDNA library of orange-spotted grouper (*Epinephelus coioides*), and revealed the dominant expression both in pituitary and testis. Moreover, we demonstrated its antibacterial activity and antiviral activity both *in vitro* and *in vivo*. Groupers, as protogynous hermaphroditic and sex reversal species [35], have been considered as good models to study sex differentiation and endocrine regulation [36], therefore, the dominant expression of pituitary and testis and the antimicrobial roles imply that the currently identified grouper β-defensin might play a pivotal role in reproduction endocrine regulation in addition to the innate host defense.

## Results

### Molecular characterization and genomic organization of grouper β-defensin

**Figure 1:**
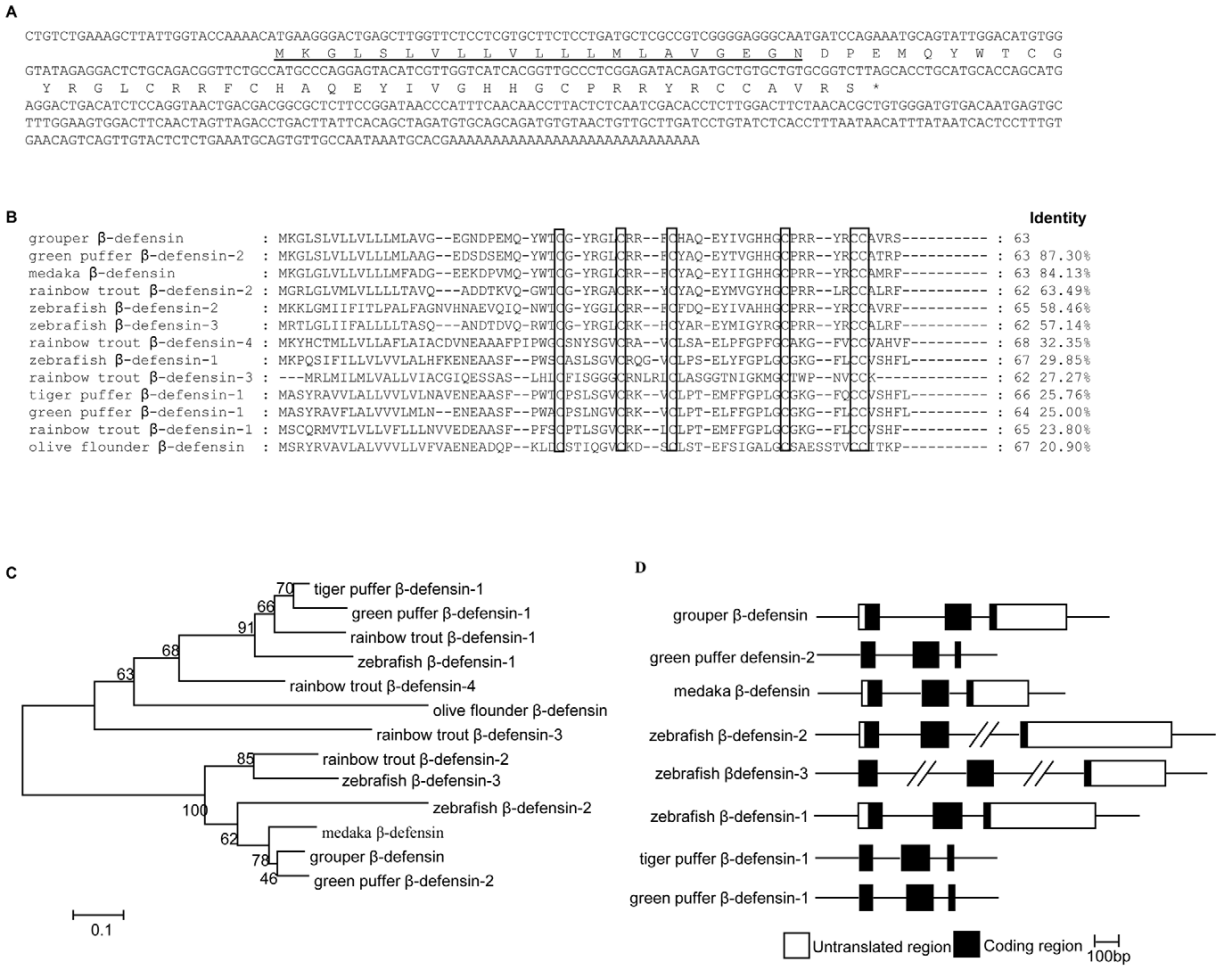
Molecular characterization of grouper β-defensin. (A) Nucleotide and deduced amino acid sequences of grouper β-defensin. The signal peptide is underlined. (B) Multiple alignment of grouper β-defensin amino acid sequences with other β-defensin proteins from fish. They are green puffer β-defensin-1 (BN000873) and green puffer β-defensin-2 (BN000874), medaka β-defensin (EU676010), zebrafish β-defensin-1 (AM181358), zebrafish β-defensin-2 (AM181359) and zebrafish β-defensin-3(AM181360), rainbow trout β-defensin-1(AM282655), rainbow trout β-defensin-2 (FM212656), rainbow trout β-defensin-3 (FM212657) and rainbow trout β-defensin-4 (FM212658), tiger puffer β-defensin-1(BN000875), olive flounder β-defensin (GQ414989). The identity values are on the right. The six conservative cystine residues are marked by black box. (C) Phylogenetic tree of grouper β-defensin peptide with other fish β-defensins. The bootstrap values were generated by testing the tree 1000 times. (D) Schematic representation of genomic structure of grouper β-defensin and other seven fish β-defensins.

A total of 103 ESTs were sequenced from the female orange-spotted grouper pituitary cDNA plasmid library, and 5 ESTs were revealed to have a high similarity with green puffer β-defensin-2 [15]. According to the EST sequences, we designed the primers and cloned the full-length cDNA of grouper β-defensin using RACE strategy. As shown in Fig. 1A, the full-length cDNA is 558 bp, consisting of a 28 bp 5′ UTR, a 338 bp 3′UTR and a 192 bp open reading frame which encodes 63aa (accession no. AY129305). The first N-terminal 21 aa is predicted as a signal peptide by signal P program (3.0 SERVER). The mature peptide contains 42 amino acid residues with a predicted molecular weight of 5.07 kDa. The theoretical isoelectric point is 8.92. And the calculated net charge is +4 at physiological environment (pH 7.4).

Multiple amino acid alignment reveals six completely conserved cysteines (C_1_, C_2_, C_3_, C_4_, C_5_, C_6_) and several highly conserved leucines (L) and glycines (G) among these fish β-defensins (Fig. 1B). The grouper β-defensin has highest (87.30%) amino acid identities to green puffer β-defensin-2, and has 84.13%, 63.49%, 58.46% and 57.14% amino acid identities to medaka β-defensin, rainbow trout β-defensin-2, zebrafish β-defensin-2 and zebrafish β-defensin-3 respectively. It has less than 30% identities to rainbow trout β-defensin-3, tiger puffer β-defensin-1, zebrafish β-defensin-1, green puffer β-defensin-1, rainbow trout β-defensin-1and olive flounder β-defensin. Phylogentic tree analysis reveals two branches. Grouper β-defensin, green puffer β-defensi-2, medaka β-defensin, zebrafish β-defensin-2, zebrafish β-defensin-3 and rainbow trout β-defensin-2 are clustered in one branch, while the other sequences form another branch (Fig. 1C).

To analyze the genomic organization of grouper β-defensin, we amplified the genomic sequence (907 bp) from the muscle genome DNA. In comparison with the mRNA sequence, grouper β-defensin gene has three exons (87, 111 and 331 bp) and two introns (297 and 81 bp) (Fig. 1D), as found in other fish defensins, including green puffer β-defensin-2, medaka β-defensin, zebrafish β-defensin-1, zebrafish β-defensin-2, zebrafish β-defensin-3, tiger puffer β-defensin-1 and green puffer β-defensin-1. Unexpectedly, the two introns in zebrafish β-defensin-3 were 2426 bp and 2108 bp, larger than those of other fish defensins. Signal peptide is localized in the first and second exon. The C_1_, C_2_, C_3_ and C_4_ are localized in the second exon, and the C_5_ and C_6_ are localized in the last exon.

### Dominant expression of grouper β-defensin both in pituitary and testis

**Figure 2:**
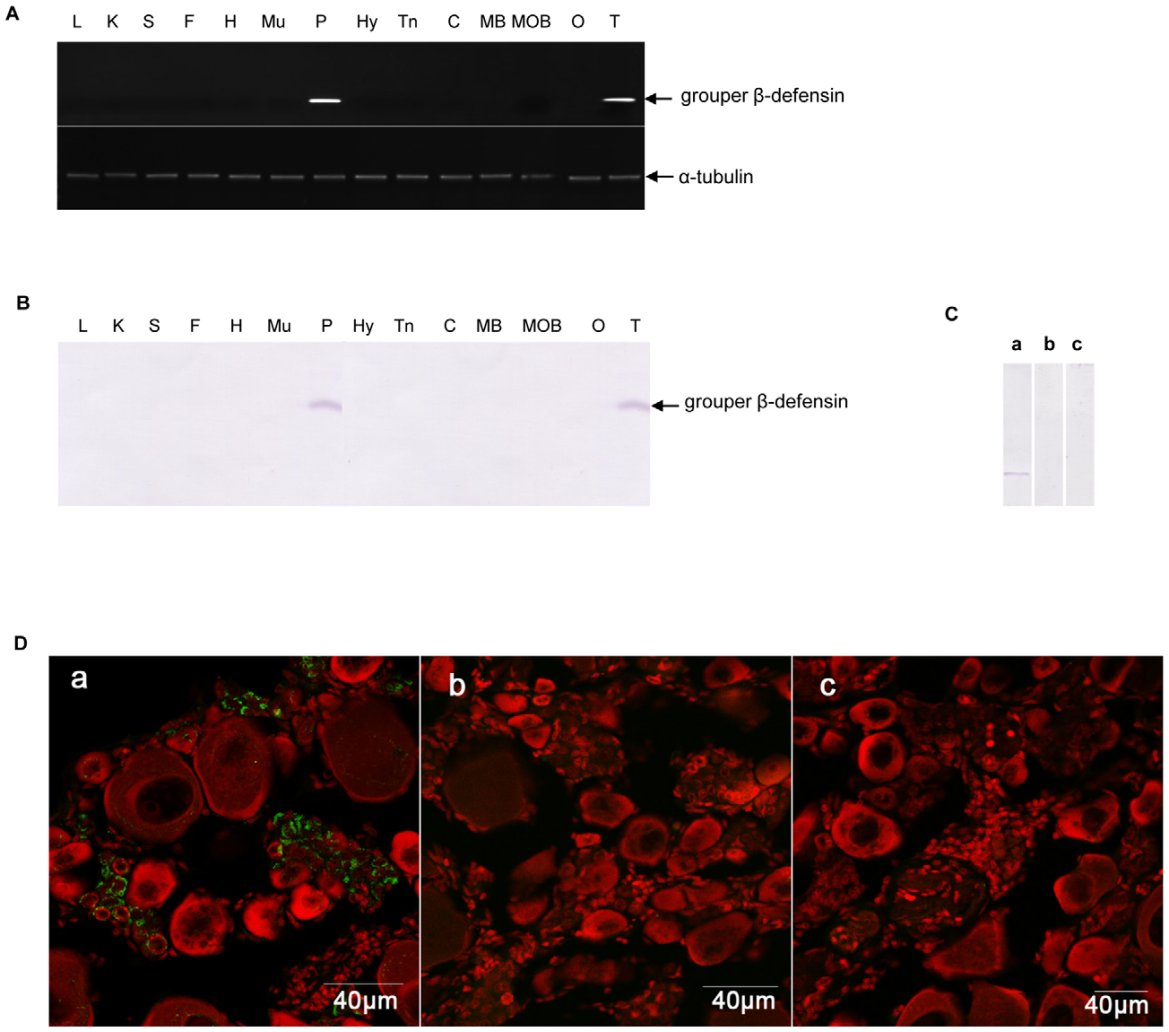
Adult tissue distribution profiles of grouper β-defensin and the specificity detection of anti-grouper β-defensin antiserum. (A) Expression pattern analyzed by RT-PCR and Western blot (B). L: liver; K: kidney; S: spleen; F: fat; H: heart; Mu: muscle; P: pituitary; Hy: hypothalamus; Tn: telencephalon; C: cerebellum; MB: midbrain; MOB: medulla oblongata; O: ovary; T: testis. α-tubulin was employed as a positive control. (C) The specificity of anti-grouper β-defensin serum detected by Western blot and immunofluorescence (D). The sections of grouper sexual reversal gonad were immunostained by the anti-grouper β-defensin serum (a), the pre-adsorbed anti-grouper β-defensin serum with extra recombinant grouper β-defensin protein (b), and the pre-immuned rabbit serum (c) respectively. Red fluorescence stained by PI indicates the cellular nucleus.

Tissue distribution profiles of β-defensin expression are very various even between closely related species [37]. In this study, we firstly analyzed the adult tissue distribution of grouper β-defensin expression by semi-quantitative RT-PCR and Western blot. As shown in Fig. 2A and 2B, the grouper β-defensin transcript (Fig. 2A) and protein (Fig. 2B) are dominantly expressed both in pituitary and testis, and no any signals are detected from other analyzed tissues, including liver, kidney, spleen, fat, heart, muscle, telencephalon, cerebellum, midbrain, medulla oblongata and ovary.

Moreover, we evaluated the specificity of the anti-grouper β-defensin polyclonal antibody by Western blot and immunofluorescence respectively. As shown in Fig. 2C, the anti-grouper β-defensin antibody specifically recognizes an about 5.5 kDa protein in the pituitary extract (Fig. 2C-a), while pre-adsorbed antiserum (Fig. 2C-b) with the purified recombinant grouper β-defensin protein or the pre-immnune serum (Fig. 2C-c) can not detect the specific 5.5 kDa polypeptide in the same pituitary extract. Immunofluorescence localization was consistent with that of Western blot. As shown in Fig. 2D, strong immunofluorescence is detected by the anti-grouper β-defensin antibody in some spermatogonia from the intersexual gonad sections of the protogynous hermaphroditic red-spotted grouper (Fig. 2D-a), whereas no immunofluorescence signal is observed when using the pre-adsorbed antiserum with the purified recombinant grouper β-defensin protein for 16 h at 4°C (Fig. 2D-b) or using the pre-immuned serum (Fig. 2D-c) as primary antiserum. The data indicate that the antibody has high specificity to the grouper β–defensin.

### Grouper β-defensin is upregulated from intersexual gonad to testis during sex reversal

**Figure 3:**
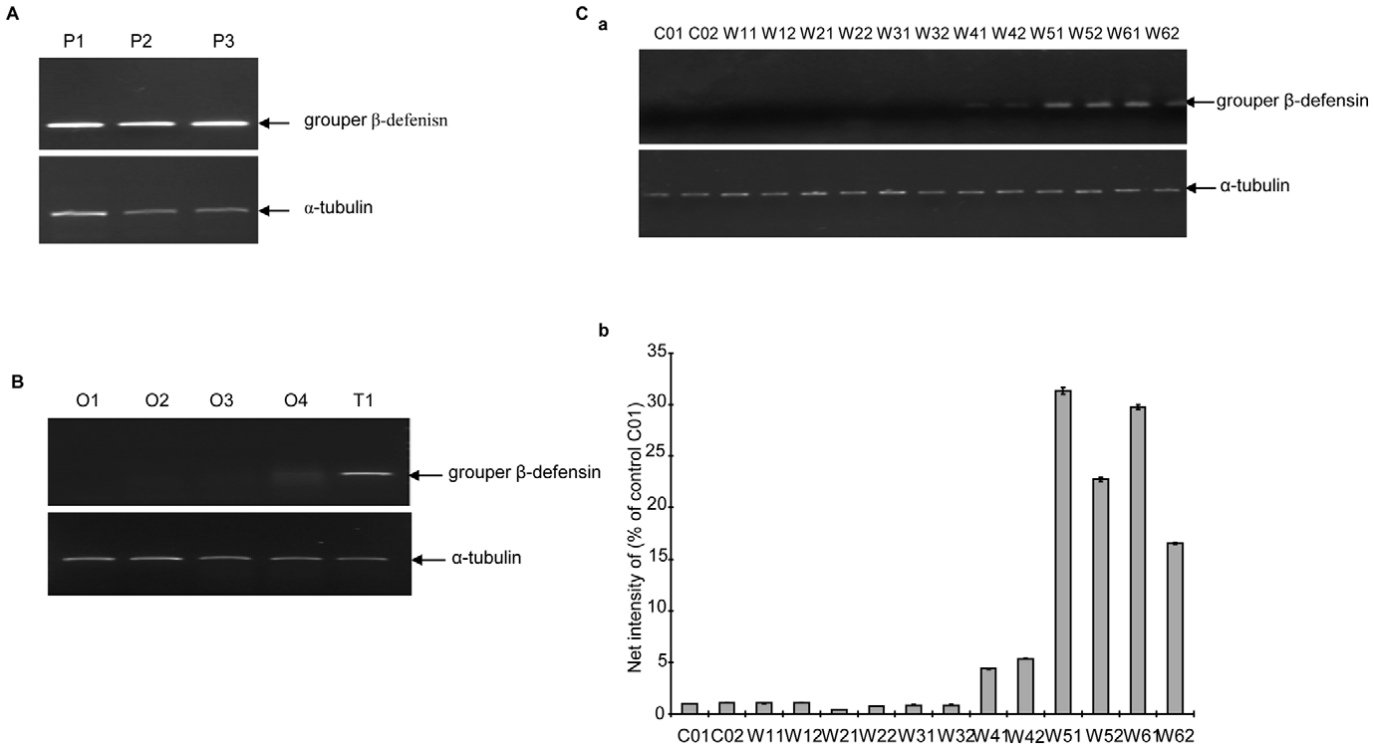
The expression of grouper β-defensin is upregulated during the period of sex transformation. (A) The expression of grouper β-defensin in the pituitaries of grouper with gonads in different stages. The total RNAs of pituitaries were isolated from grouper with undevelopmental, mature ovary and mature testis respectively. (B) Differential expression of grouper β-defensin in different stage gonads of red-spotted grouper. The total RNAs of gonads were respectively isolated from the different gonad stages with different sizes: O1: immature ovary from 150 g body weight; O2: ovary with previtellogenic oocytes from 450 g body weight; O3: ovary with vitellogenic oocytes from 700 g body weight; O4: ovary with vitellogenic oocytes from 950 g body weight; T1: testis from 1600 g body weight. α-tubulin was used as control. (C) Expression of grouper β-defensin in different stage gonads of red-spotted grouper during artificial sex inversion. (a) RT-PCR analysis of grouper β-defensin expression. α-tubulin was amplified at the same conditions as a positive control in each sample. (b) The grouper β-defensin mRNA intensities as shown in (a) were analyzed by Band Leader Applification Software Ver. 3.0. Values represent the means ± S.D. of three separate experiments. C01 and C02: the gonads from two individuals before sex inversion experiment, W11, W12, W21, W22, W31, W32, W41, W42, W51, W52, W61 and W62: the gonads from two individuals after feeding with MT for week 1–6, respectively.

In normal physiological conditions, several mammal defensins are sensitive to androgen and are developmentally regulated [38], [39]. To reveal the association of grouper β-defensin expression with sex reversal, we firstly evaluated the transcript level in the pituitaries and gonads of groupers at different stages. As shown in Fig. 3, the grouper β-defensin transcript level is always abundant in the pituitaries of different stage red-spotted groupers with un-development gonad, mature ovary and mature testis (Fig. 3A), but the β-defensin transcript level is very various in the different stage gonads. Abundant grouper β-defensin transcript is observed in the testis of 1600 g-weight male, and only a few of transcript is detected in the gonad of 950 g-weight maturing female, whereas no any transcript is found in the gonads of 150 g-weight immature female, 450 g-weight developing female, and 700 g- weight maturing female (Fig. 3B). Previous anatomical and microscope observations have shown that the 1600 g-weight individual is mature male with testis containing sperms, whereas others were females with ovaries at different stages of oogenesis [40], [41].

For the artificial sex reversal groupers, the total RNAs were isolated from the gonad tissues of two individuals that were randomly sampled in the MT feeding group [42] at weekly intervals, and were used for RT-PCR analysis. As shown in Fig. 3C, the grouper β-defensin starts to transcribe at the 4th week after MT feeding, and reaches to a peak level at the 5th week. The high up-regulation transcription level suggests that the grouper β-defensin might have a significant role in testis development and spermatogenesis.

### Sequence characterization and responsible activity regions of grouper β-defensin promoter

**Figure 4:**
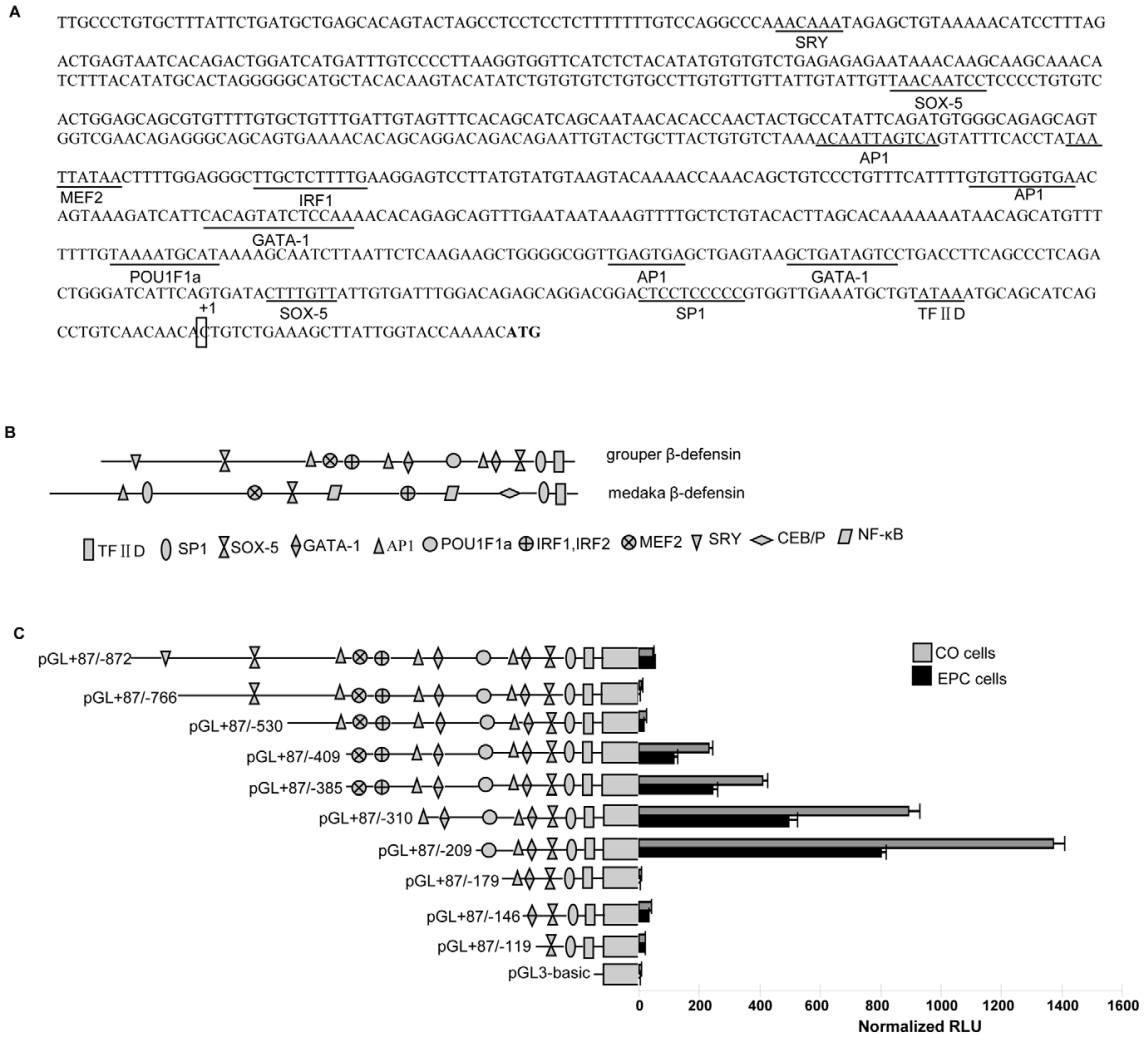
Map and deletion analysis of grouper β-defensin promoter. (A) Sequence of grouper β-defensin promoter region. Consensus nucleotide sequences corresponding to potential transcription binding sites are underlined and labeled. (B) Schematic diagrams and comparison of potential transcription factor binding sites between grouper β-defensin promoter and medaka β-defensin promoter. (C) The progressive 5′ deletion series and their luciferase activities in CO (grey bars) and EPC (black bars). pRL-TK was used as internal control. The promoter activity is presented as relative light units (RLU) normalized to Renilla luciferase activity. The data shown are derived from a representative experiment reported as the mean (n = 3) ± SD.

A 900 bp 5′-flanking sequence of grouper β-defensin was isolated from the grouper genome DNA. It includes a 28 bp non-coding region and a 872 bp putative promoter. The consensus TATA box motif is in the 25 bp upstream of the transcription start site. A total of 13 transcription binding sites, such as transcription factor II (TFIID), stimulating protein 1(SP1), activator protein 1(AP1), sry-related HMG box 5 (SOX5), interferon regulatory factor-1 (IRF-1), myeloid elf-1 like factor -2 (MEF-2), GATA binding factor-1 (GATA-1), sex-determining region Y (SRY) and pituitary specific pou domain transcription factor 1a (POU1F1a) [43], were predicted in the isolated grouper β-defensin promoter by TESS and MatInspector (Fig. 4A). In comparison with the reported medaka β-defensin promoter (19), the grouper β-defensin promoter has a pituitary-specific POU1F1a transcription binding site, and does not contain NF-κB binding site (Fig. 4B).

To reveal the responsible regions of grouper β-defensin promoter activity, series 5′ deletion mutant constructs were constructed in pGL3-basic vector as described previously (19), and used to transiently transfect *Ctenopharyngodon idellus* ovary (CO) cells and epithelioma papulosum cyprini (EPC) cells (44). As shown in Fig. 4C, a peak activity appears in the −209/+87 construct, and the normalized relative light units (RLU) exceeds 800, whereas in the +87/−179, the activity is the same to the control pGL3-basic. The data indicate that major responsible region of grouper β-defensin promoter activity ranges from −180 to −208. Therefore, the POU1F1a binding site in this region might be important for the pituitary-specific expression of grouper β-defensin.

### Pituicyte localization of grouper β-defensin in pituitary

**Figure 5:**
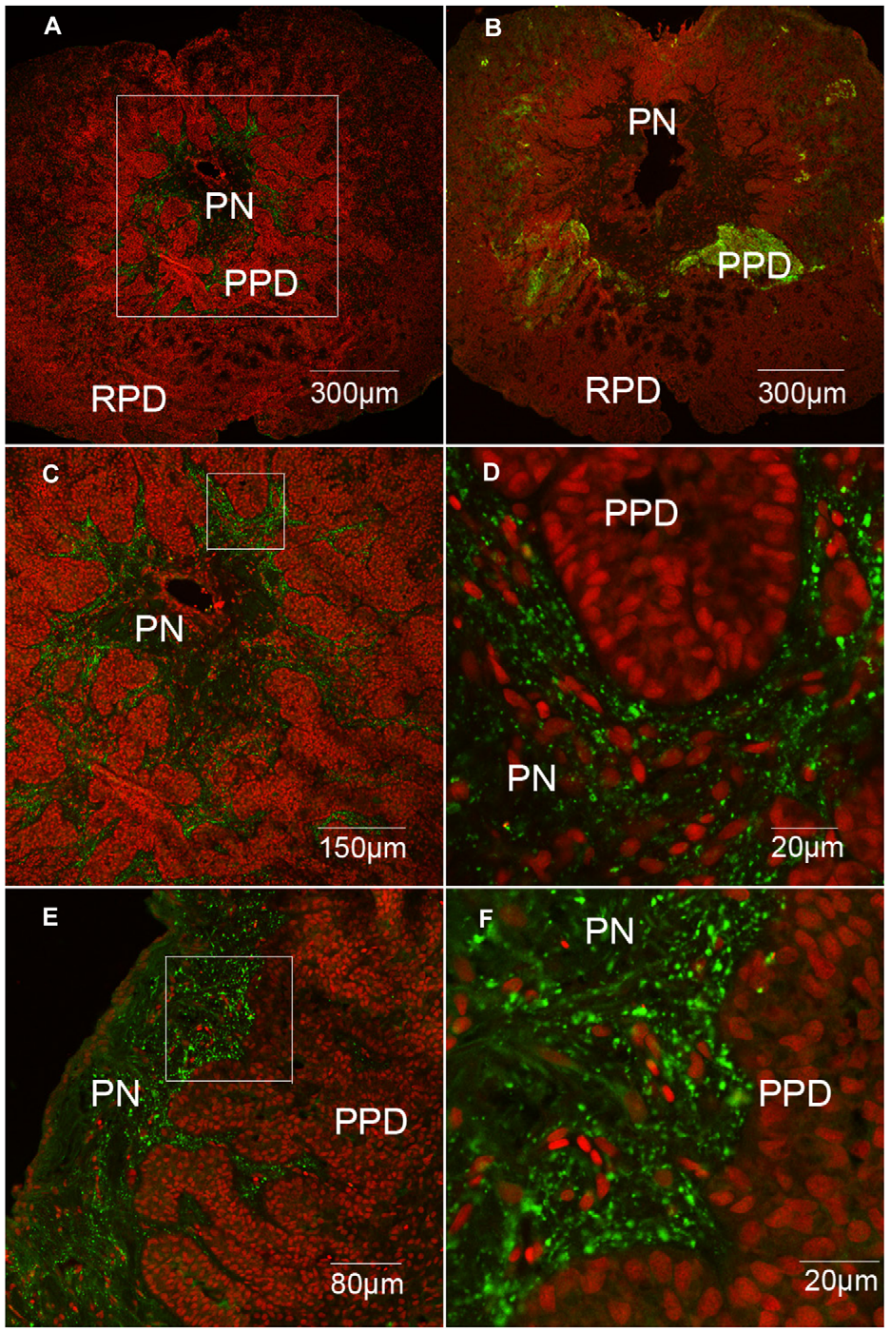
Immunofluorescence detection of grouper β-defensin protein (green) in grouper pituitary. (A) TSH signals in the anterior pituitary. (B) Grouper β-defensin signals in the posterior pituitary of transversal section of pituitary, and (C-D) are higher magnifications of positive signal area in B. (E) Grouper β-defensin signals in the posterior pituitary of sagittal section of pituitary. (F) is higher magnification of positive signal area in E. RPD:rostral pars distalis; PPD: proximal pars distalis; PN: pars noversa. Red fluresecence was stained by Propidium Iodide for cellular nucleus.

Following the above investigation, we used the anti-grouper β-defensin antibody to trace the expressed cells and distribution in the grouper pituitary, and Propidium Iodide staining for cellular nucleus was performed. As shown in Fig. 5, in comparison with strong TSH signal in the proximal pars distalis (PPD) [45] (Fig. 5A), the grouper β-defensin immunofluoresence signal is observed only in the neurohypophysis from the transversal section of grouper pituitary, and no any signal appears in the adenohypophysis, including proximal pars distalis (PPD) and rostral pars distalis (RPD) (Fig. 5B). And, higher magnification further revealed detail distribution characterization of the grouper β-defensin immunoreactive signal (Fig. 5C and 5D). The immunoreactive signals are scattered throughout the neurohypophysis, but it seems stronger in the posterior of *pars nervosa* than those of the anterior neurohypophysis. In the sagittal pituitary section, the positive cells are long and slender, resembling filament, in which cytoplasmic projections often extend between the nerve fibers (Fig. 5E and 5F). Obviously, the grouper β-defensin-expressed cells should be pituicytes, the typical cells in pituitary neurohypophysis, which implies the pivotal regulation role of grouper β-defensin in the pituitary.

### Spermatogonium-specific expression of grouper β-defensin in testis

**Figure 6:**
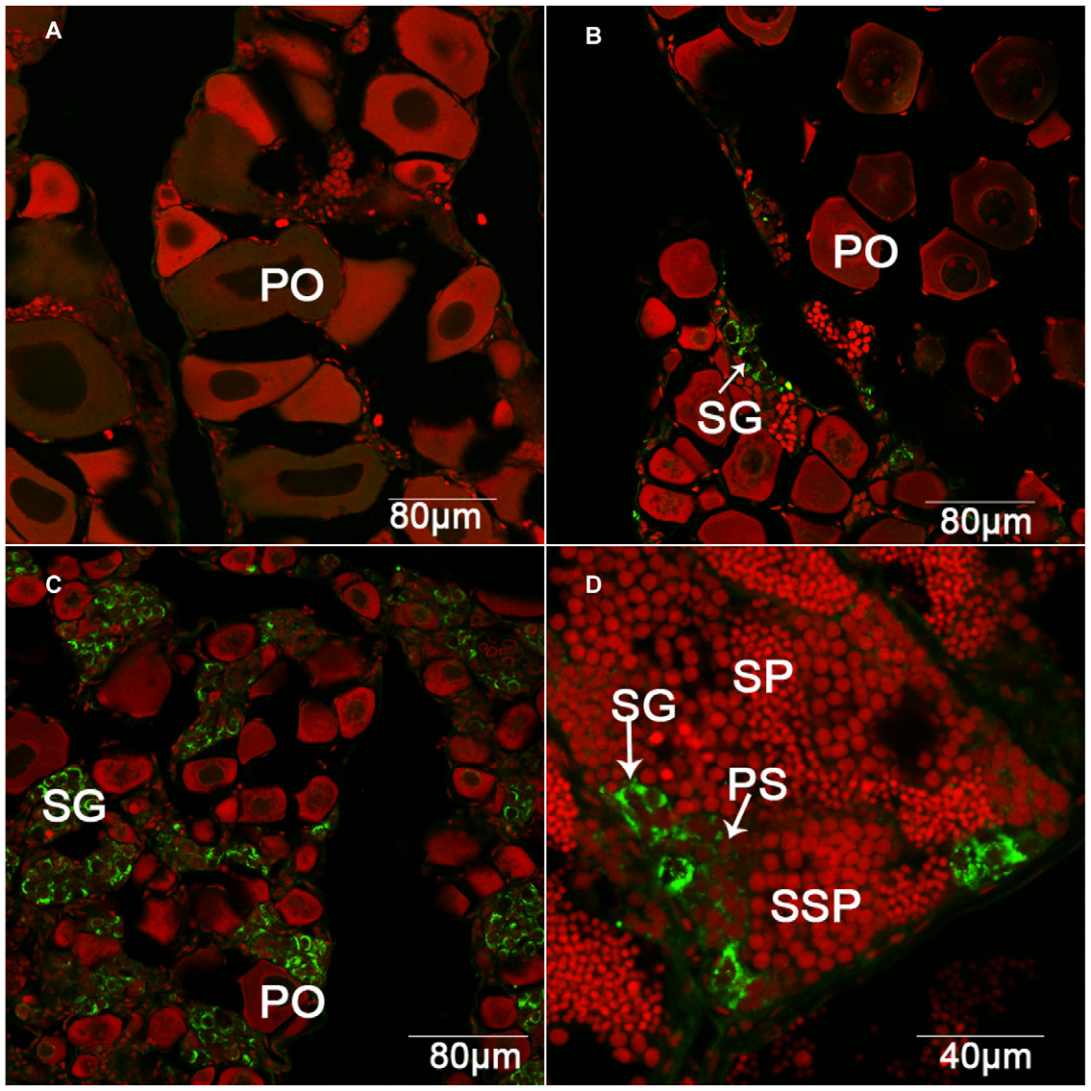
Spermatogonium-specific immunofluorescence localization of grouper β-defensin protein (green) in the grouper gonads. (A) Ovary section; (B) Gonad section at the early sex reversal stage; (C) Gonad section at intersex stage; (D) Testis section. PO: previtellogenic oocyte; SG: spermatogonia; PSP: primary spermatocytes; SSP: secondary spermatocytes; SPZ: spermatozoa. Red fluresecence was stained by Propidium Iodide for the nuclei.

Subsequently, we investigated dynamic changes and cell localization of the grouper β-defensin by immunofluorescence in the grouper gonads at different stages from ovary to testis in the natural sex reversal process. As shown in Fig. 6, in the gonad of maturing female, which contains a lot of vitellogenic oocytes, the grouper β-defensin expression can not be detected by immunofluorescence (Fig. 6A). When the grouper initiates to sex reversal, the gonad begins to appear early transitional characters. The cysts of male germ cells, from spermatogonia to spermatozoa, appear in the edge of lobules. Strong grouper β-defensin immunofluorescence signal is observed in the cytoplasm of spermatogonia (Fig. 6B). Following further sex reversal, oocytes reduce largely in number, and along with spermatogonium increase, the grouper β-defensin expression level increases rapidly (Fig. 6C). When the gonad completely changes to testis, the strong grouper β-defensin immunofluorescence is still detected in the spermatogonia, and some residual grouper β-defensin fluorescence is observed in primary spermatocytes (Fig. 6D). The data indicate that grouper β-defensin is expressed by spermatogonia in testis, suggesting that it might play an important role in sex reversal and spermatogenesis.

### Antibacterial activity of recombinant grouper β-defensin specific to Gram-negative bacteria

Recombinant plasmids pET-28-grouper β-defensin was transformed into *E. coli* BL21. After IPTG (0.8 mM) induction for 4 h, the recombinant grouper β-defensin proteins (9 kDa) were expressed mostly in the insoluble fractions, but not in the supernatants of bacterial cells after disruption by sonication. After purified by the denaturing conditions, more than 95% purity recombinant grouper β-defensin protein was obtained. The yield of the purified grouper β-defensin protein was about 5 mg/L broth.

**Figure 7:**
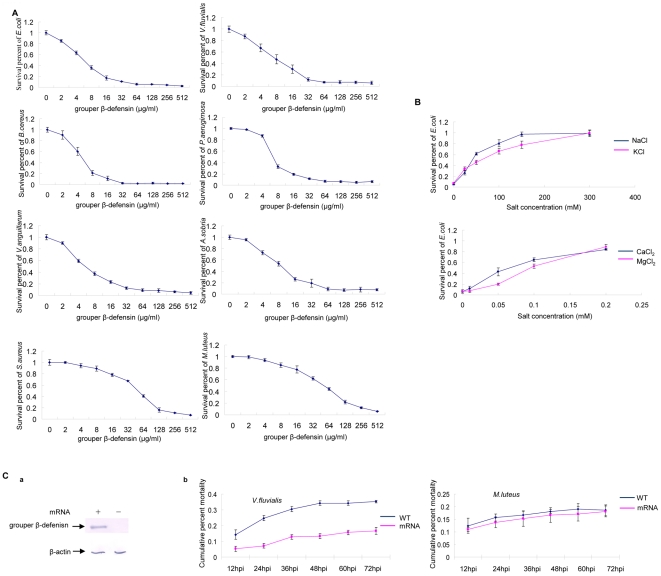
Antimicrobial activity of grouper β-defensin. (A) Antimicrobial activities of grouper β-defensin *in vitro* to eight different strains (shown in respective chart). X-axis indicates the protein gradient concentration (µg/ml) and the Y-axis shows the survival rate (means ± SD). At least three independent experiments were conducted. (B) Effects of NaCl, KCl, CaCl_2_ and MgCl_2_ concentration on the antimicrobial activity of grouper β-defensin. *E.coli* were incubated for 12 h with 64 µg/ml grouper β-defensin in the culture medium containing various concentration ions. Values shown are mean ± SD. (C) Antimicrobial activity of grouper β-defensin *in vivo*. (a) Western blot was employed to detect the expression of grouper β-defensin by embryos. (b) At 24hpf, the control embryos injected with pure water and embryos injected grouper β-defensin mRNA were challenged with the Gram-negative bacterium *Vibrio flurialis* and Gram-positive bacterium *Micrococcus luteus*, respectively. The survival rates were recorded at the indicated times after infection. The experiments were repeated three times with 70 embryos per group.

Since grouper β-defensin has very high identity to green puffer β-defensin and medaka β-defensin, we firstly used the purified recombinant grouper β-defensin protein to perform antibacterial analysis in eight strains of bacteria, including six Gram-negative strains (*E. coli*, *V. fluvialis*, *B. cereus*, *P. aeruginosa*, *V. anguillarum* and *A. sobria*) and two Gram-positive strains (*S. aureus* and *M. luteus*). Similarly to medaka β-defensin, the purified recombinant grouper β-defensin also exhibited higher antibacterial activity to Gram-negative bacteria than to Gram-positive bacteria. Under high concentration of 512 µg/ml, the survival rates of all eight strains were lower than 10%, but in comparison with Gram-positive strains, lower concentration was sufficient to resist against Gram-negative strains (Fig. 7A). Moreover, we calculated the protein concentrations that killed 50% (vLD_50_) and 90% bacteria (vLD_90_). As shown in Table 1, the vLD_50_ (5.1±0.4 to 8.8±0.5) and vLD_90_ (17.5±4.0 to 59.4±8.8) in Gram-negative strains were much lower than that in Gram-positive strains (vLD_50_ from 53.1±2.1 to 53.9±4.7, vLD_90_ from 296.5±65.5 to 358.5±46.5). The data indicated that grouper β-defensin should have more strong effect to kill Gram-negative bacteria than to kill Gram-positive bacteria.

**Table 1:** Antimicrobial activities of grouper β-defensin against two Gram-positive and six Gram-negative strains.

Test organism	vLD_50_ (µg/ml [mean ± SEM])	vLD_90_ (µg/ml [mean ± SEM])
*E. coli*	6.7±1.3	39.0±3.7
*V. fluvialis*	7.4±1.0	43.0±10.0
*B. cereus*	5.1±0.4	17.5±4.0
*P. aeruginosa*	6.7±0.2	44.5±11.8
*V. anguillarum*	5.7±0.6	58.2±23.4
*A. sobria*	8.8±0.5	59.4±8.8
*M. luteus*	53.9±4.7	296.5±65.5
*S. aureus*	53.1±2.1	358.5±46.5

β-defensins have been shown to be inactivated at high salt concentrations[46]. To determine whether antibacterial activity of grouper β-defensin is similarly affected, salt sensitivity assays were implied. Since 64 µg/ml purified recombinant grouper β-defensin proteins can almost fully inhibit the growth of *E.coli*, we used this concentration for the later salt effect examinations. The concentration of Na^+^, K^+^, Ca^2+^ and Mg^2+^ were adjusted with NaCl, KCl, CaCl_2_ and MgCl_2_, respectively. As shown in Fig. 7B, the bactericidal activity was suppressed by the increasing concentrations of all salts. The antibacterial activity of grouper β-defensin was substantially diminished at high concentration of NaCl (150 mM) and KCl (300 mM). In comparison, the antimicrobial activity of grouper β-defensin was more sensitive to CaCl_2_ and MgCl_2_. The ability of grouper β-defensin to inhibit growth of *E. coli* diminished about fifteen fold when divalent cations concentrations were increased from 0 mM to 0.2 mM.

To investigate the antibacterial activity of grouper β-defensin *in vivo*, we injected grouper β-defensin mRNA or pure water into rare gudgeon embryos, respectively. Then the two groups of embryos were exposed to pathogen bacterium *V. flurialis* or *M. luteus* by static immersion. Similar to the results *in vitro*, grouper β-defensin has more strong effect to kill Gram-negative bacterium *V. flurialis* than to kill Gram-positive bacterium *M. luteus*. As shown in Fig. 7C, the expressed β-defensin protein can be detected by Western blot in the embryos injected with β-defensin mRNA, and the survival rates are about twice higher in the grouper β-defensin mRNA injected embryos than in the control wild-type embryos when the two groups of embryos are infected by *Vibrio flurials*, whereas no any significant difference is observed when the two group embryos are infected by *Micrococcus luteus*.

### Antiviral activity of grouper β-defensin against an iridovirus infection

**Figure 8:**
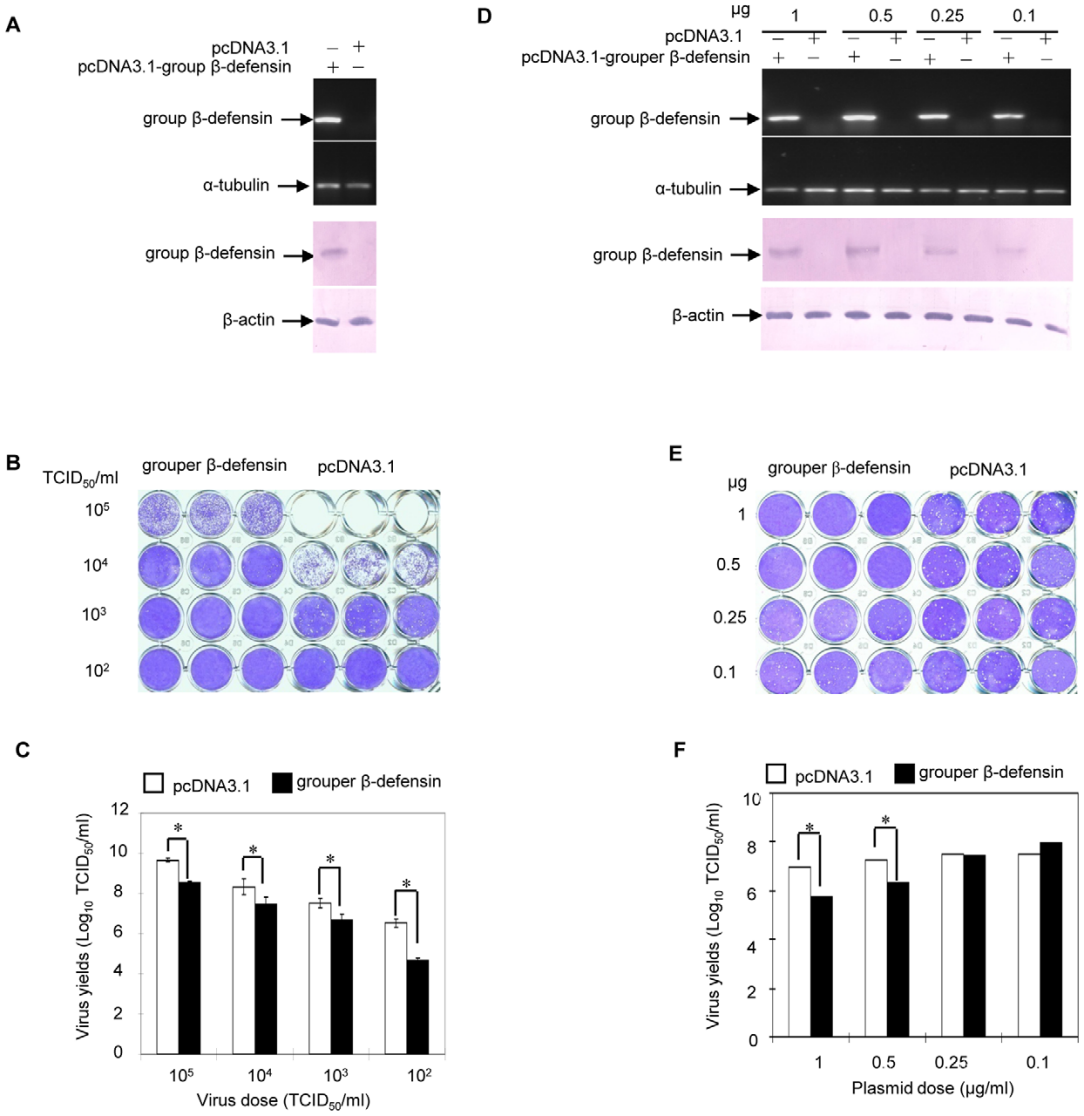
Antiviral activity of grouper β-defensin. (A) EPC cells seeded in 24-well plates were transfected for 24 h with 0.5 µg of pcDNA3.1-grouper β-defensin or pcDNA3.1 as control. Grouper β-defensin mRNA and the protein was detected by RT-PCR and Western blot respectively, and (B) the other group of transfected was challenged with RGV with different dose (10^5^ TCID_50_/ml, 10^4^ TCID_50_/ml, 10^3^ TCID_50_/ml and 10^2^ TCID_50_/ml), respectively. 48 h later, cells were then stained with crystal violet for detection of CPE, and (C) the culture supernatants were collected to detect the virus titers. (D) EPC cells seeded in 24-well plates were transfected for 24 h with pcDNA3.1-grouper β-defensin or pcDNA3.1 at dose of 1 µg, 0.5 µg, 0.25 µg and 0.1 µg, respectively. Grouper β-defensin mRNA and the protein was detected by RT-PCR and Western blot respectively, and (E) the other of transfected cells was challenged with RGV at multiplicity of infection 10^3^ TCID_50_/ml. 48 h later, cells were then stained with crystal violet for detection of CPE, and (F) the culture supernatants were collected to detect the virus titers. The data shown is a representative of three independent experiments. Differences between control cells and grouper β-defensin transfected cells are significant *p<0.05.

Moreover, we analyzed the antiviral role of grouper β-defensin in cells by transfection assays. Firstly, EPC cells were transfected for 24 h with 0.5 µg of grouper β-defensin construct pcDNA3.1-grouper β-defensin or empty vector (pcDNA3.1) as control, and the successful transfection was confirmed by RT-PCR and Western blot detection of grouper β-defensin mRNA and protein (Fig. 8A). Then the transfected cells were infected with 10^5^, 10^4^, 10^3^ and 10^2^ TCID_50_/ml of an iridovirus *Rana grylio* virus (RGV) [47], respectively. When obvious cytopathogenic effect (CPE) was produced for 48 h incubation in the control cells transfected with empty vector pcDNA3.1, significantly, only less CPE was found in the pcDNA3.1-grouper β-defensin-transfected cells (Fig. 8B). Fig. 8C shows the virus yields between the two group cells under different virus dose infection, and the virus yields in the pcDNA3.1-grouper β-defensin-transfected cells are significantly lower than that in the control cells transfected with empty vector pcDNA3.1.

Furthermore, EPC cells were transiently transfected with different doses of grouper β-defensin construct or empty vector (pcDNA3.1), and the expressed β-defensin mRNA and protein were confirmed by RT-PCR and Western blot detection (Fig. 8D). Following the infection of transiently transfected cells with 10^3^ TCID_50_/ml RGV, CPE was observed, and the viral yields were determined. As shown in Fig. 8E, almost no CPE is found in cells transfected with 1 µg or 0.5 µg of grouper β-defensin construct. In contrast, obvious CPE is observed in control cells that were transfected with empty vector pcDNA3.1. Additionally, virus yield estimation also revealed significant differences between the two group cells. In control cells, the difference of virus yields was slight when the cells were transfected with 1 µg, 0.5 µg, 0.25 µg and 0.1 µg empty vector pcDNA3.1. In the pcDNA3.1-grouper β-defensin transfected cells, there was a gradual elevation in virus titers along with the decrease of the trasfected plasmid concentration (Fig. 8F). The results together indicate that grouper β-defensin has antiviral role in against the RGV iridovirus infection.

## Discussion

A significant and intriguing finding in this study is the dominant expression of grouper β-defensin both in pituitary and testis. The first vertebrate β-defensin was identified in bovine tracheal mucosa [48]. Since then, more and more β-defensins were isolated from other vertebrates, such as human [49], mouse [2], avian [12] and fish [15]. Generally, the tissue distribution of β-defensins is extensive and diverse. These molecules are mostly produced by tissues involved in host defense [26]. In the current study, we cloned and characterized a β-defensin from the pituitary SMART cDNA library of grouper, and found that the β-defensin is simultaneously expressed both in the grouper pituitary and testis (Fig. 3). It was well known that hypothalamus-pituitary-gonad axis is an important neuro-endocrine system for regulating vertebrate reproduction and sex differentiation [50]. Several β-defensins had been detected in brain [33], [34] or male reproductive tract [29], [39], but the expression both in pituitary and testis is the first finding. Some epididymis-specific β-defensins also possess other function more than antimicrobial activity. For examples, Bin1b, a natural epididymis-specific β-defensin in rat, was demonstrated to be important for the sperm mature in rat [23], [29]. ESP13.2, produced by macaque epididymis, was confirmed to have a function in the sperm capacitation process [51]. Therefore, the dominant expression of grouper β-defensin both in pituitary and testis implies that the grouper β-defensin might play a significant role in the endocrine regulation and sex differentiation.

Moreover, promoter sequence and the responsible activity region analyses revealed the pituitary-specific POU1F1a transcription binding site and testis-specific SRY responsible site in the grouper β-defensin promoter, and demonstrated that the pituitary-specific POU1F1a transcription binding site was major responsible region of grouper β-defensin promoter activity (Fig. 4). However, the POU1F1a is a pituitary-specific transcription factor, it could not be expressed in the CO and EPC cells. Significantly, the POU1F1a has been revealed to belong to the POU domain transcription factor family that includes POU1F1, Oct-1, and Oct-2, and these factors share a highly conserved bipartite DNA-binding domain. And, Oct-1 has been demonstrated as a ubiquitously expressed factor [52]. Therefore, it is suggested that the ubiquitous Oct-1 should be expressed both in CO cells and EPC cells, and the high promoter activity might be resulted by the binding of the ubiquitous Oct-1 and the POU1F1a site.

According to a previous study from our laboratory, NF-κB binding site was important for medaka β-defensin response to LPS [19]. And, this binding site was also crucial for inducing human β-defensin 2 by LPS in RAW264.7 cells [53]. Furthermore, we used anti-grouper β-defensin serum to localize pituicyte expression in pituitary (Fig. 5) and spermatogonium expression in testis (Fig. 6). Unlike other β-defensins widely distributed in many tissues [15], [16], [19], the expression of grouper β-defensin in grouper pituicytes and spermatogonium might constitute an essential component in maintaining the endocrine regulation and sex reversal process.

Most β-defensins have been demonstrated to have antimicrobial activity [54]. Some of them, such as mouse β-defensin 1 [2] and human β-defensin 3 [55], can kill both Gram-positive and Gram-negative bacteria. Some are specific to Gram-negative bacteria, such as human β-defensin 1 [46], human β-defensin 2 [56] and medaka β-defensin [19]. In this study, we demonstrated that the grouper β-defensin have the antibacterial activity-specific to Gram-negative bacteria, and the activity ability reduces along with the increase of salt concentration (Fig. 7), which is similar to other β-defensins, such as mouse BD-1[2] and human BD-1[46]. Additionally, the grouper β-defensin also exhibits antiviral activity against RGV, an iridovirus. Although there are many reports on β-defensin against virus infection [57], [58], in fish, only Falco et al. demonstrated that rainbow trout β-defensin-1 exhibited antiviral activity against viral haemorrhagic septicaemia rhabdorvirus (VHSV) [16]. Therefore, the dual roles for antibacterium and antivirus imply that the grouper β-defensin might play significant roles in immunity defense of reproduction endocrine regulation.

In conclusion, we have identified and characterized a fish β-defensin from the protogynous hermaphrodite grouper, revealed its dominant expression both in pituitary and testis, and demonstrated the dual roles for antibacterium and antivirus. The expression pattern in reproduction axis and the dual roles for antibacterium and antivirus might be related to the sex reversal in the grouper fish. Perhaps, our current findings open the door for understanding the reproduction and endorcrine regulation roles of β-defensin in fish, although this issue remains to be investigated more clearly.

## Materials and Methods

### SMART cDNA synthesis, grouper β-defensin identification and sequence analysis

Total RNA of pituitary was isolated from 4-year-old orange-spotted grouper by SV Total RNA Isolation System (Promega). SMART cDNAs were synthesized and amplified using the Switching Mechanism At 5′-end of RNA Transcript (SMART) cDNA Library Construction Kit (Clontech) as described previously [19], [40], [42], [59]. The plasmid cDNA library was plated to appropriate density to pick individual colonies. By screening 103 clones, five clones showing conserved β-defensin characteristic were sequenced. Signal peptide was predicted by SignalP 3.0 server (http://www.cbs.dtu.dk/services/SignalP/). The isoelectric point (PI), molecular weight (MW) and the net charge were calculated by ProtParam (http://www.expasy.ch/tools/). ClustalW (http://align.genome.jp/) was used to align multiple fish defensins, and the identity percentages were generated by Bioedit. A phylogenetic tree was constructed by using the Neighbour-Joining (NJ) algorithm based on the deduced amino acid sequences.

The genomic DNA fragment of grouper β-defensin was amplified from the total genomic DNA isolated from orange-spotted grouper muscle as described previously [60]. The genomic sequences were obtained in ensemble database (http://www.ensembl.org). All putative promoters were predicted by the 1999 Neural Network Promoter Prediction (NNPP version 2.2) (http://www.fruitfly.org/seq_tools/promoter.html). The potential transcription factor binding sites were identified by TESS and MatInspector.

### RNA isolation, reverse transcription and semi-quantitative RT-PCR

Total RNAs of liver, kidney, spleen, fat, heart, muscle, pituitary, hypothalamus, telecephalon, cerebellum, midbrain and medulla oblongata were isolated from 1-year-old orange-spotted grouper with immature ovary using SV Total RNA Isolation System according to the manufacturer's instructions (Promega). Total RNAs of pituitaries were isolated from grouper with undeveloped gonads, mature ovary and mature testis respectively. Total gonad RNAs were isolated from red-spotted grouper with body weight of 150, 450, 700, 950 and 1600 g, respectively. Total RNAs of gonads at the different artificial sex inversion stages were isolated from 14 individuals of red-spotted grouper [42]. Then RNAs were respectively reverse-transcribed with M-MLV Reverse Transcriptase (Promega) and oligo(dT)8–12 (Promega) as described by the manufacturer. According to the obtained nucleotide sequences, one pair of primers (BDF1/BDR1, Table 2) were synthesized (Sangon, Shanghai) and used to analyze adult tissue distribution and temporal expression in gonads at different developmental stages. The semi-quantitative RT-PCR was performed in a volume of 25 µl at the optimal conditions as follows: 94°C for 4 min, 94°C for 30 s, 60°C for 30 s, and 72°C for 30 s for 32 cycles followed by 72°C for 5 min. β-tubulin (tubulin-F/tubulin-R, Table 2) was amplified to provide a semi-quantitative control for PCR reaction efficiency under the same reaction conditions as grouper β-defensin.

**Table 2:** Primers used in this study.

Primers	Sequence(5′-3′)
CDSP	ATTCTAGAGGCCGAGGCGGCCGACATG-d(T)_30_ *N*−_1_ *N*
SMART II P	AAGCAGTGGTATCAACGCAGAGTGGCCAT-TACGGCCGGG
BDF1	GAATTCATATGAAGGGACTGAGCTTGGTTC
BDR1	GCTCGAGCTAAGACCGCACAGCACAGC
tubulin-F	GTGCACTGGTCTTCAGGGGTT
tubulin-R	GGGAAGTGGATGCGTGGGTAT
BDF2	GCTAGCGATCCGGAAATGCAGTATTGGAC
BDR2	CTCGAGGCTGCGCACGGCGCAGCA
BDF3	CTCGAGACCATGGATCCAGAAATGCAGTAT
BDR3	GGATCCCTAAGACCGCACGCAC
GSP1a	CCCTCCCCGACGGCGAGCATCAGGAGAAGC
GSP1b	GAAGCACGAGGAGAACCAAGCTCAG
GSP2a	GTTTTCACTGCTGCCCTCTGTTCGAC
GSP2b	TCGACCACTGCTCTGCCCACATCTGAATA
AP1	GTAATACGACTCACTATAGGGC
AP2	ACTATAGGGCACGCGTGGT
ProF1	GAGCTCCAGCCCTCAGACTGGGATCATTCAGT
ProF2	GAGCTCGCTGAGTAAGCTGATAGTCCTGACCT
ProF3	GAGCTCCTTAATTCTCAAGAAGCTGGGGCGGT
ProF4	GAGCTCATGTTTTTTTGTAAAATGCATAAAAGCAATCTT
ProF5	GAGCTCGTGTTGGTGAACAGTAAAGATCATTCAC
ProF6	GAGCTCTTGGAGGGCTTGCTCTTTTGAAGGAGT
ProF7	GAGCTCTTGGAGGGCTTGCTCTTTTGAAGGAGT
ProF8	GAGCTCAACACACCAACTACTGCCATATTCAGATG
ProF9	GAGCTCTCACAG ACTGGATCATGATTTGTCCC
ProF10	GAGCTC TTGCCCTGTGCTTTATTCT
ProR	CTCGAGCCCTCCCCGACGGCGAGCA

To further analyze the expression differences of grouper β-defensin at different stages of artificial sex reversal, image analysis was used to measure the intensity of the RT-PCR products by the software package GeneSnap (MwLibrary, Genius).

### Recombinant grouper β-defensin expression and antiserum preparation

Primers BDF2 and BDR2 (Table 2) were synthesized to generate a sequence for encoding the mature β-defensin peptide, and then the generated sequence was inserted into the expression vector pET-28a (+). After the construct was transformed to competent BL21 (DE3) *E. coli* cells and the positive clones were selected on Luria Bertani (LB) medium containing 50 µg/ml ampicillin, recombinant protein could be induced to over-express in the *E. coli* by 0.8 mM isopropyl-1-thio-β-D-galactoside (IPTG) for 4 h at 37°C. Because it formed insoluble inclusion bodies during expression, the recombinant grouper β-defensin protein was not lethal for the *E. coli*. After it was purified with His-tag purification kit (Novagene, USA), the recombinant protein was refolded by dialysis against 20 mM Tris-HCl buffer (PH 7.4) 4 times at 4°C. The disulphide bonds-related issues and the antimicrobial activity can be solved by the dialysis as described previously [61]. Tricine-SDS-PAGE was carried out to assess the purity of recombinant protein. The concentration of recombinant grouper β-defensin was measured by the BCA Protein Assay Kit (Pierce, USA) as per the manufacture's direction. Because no any antimicrobial activity difference was revealed between the recombinant defensin proteins with his-tag and without his-tag by previous studies [61], [62], the recombinant grouper β-defensin protein with a N-terminal his-tag was directly used as antimicrobial activity assays.

To acquire polyclonal antiserum, the recombinant proteins were applied to immunize white rabbit as described previously [40], [41], [56]. The specificity of the antiserum was evaluated by Western blot and immunofluorescence. Two controls were designed to determine polyclonal antibody specificity. In one control the grouper β-defensin antisera were replaced with pre-immune serum respectively. In another control the grouper β-defensin antisera was replaced by antisera pre-adsorbed with purified recombinant grouper β-defensin for 16 h at 4°C. The preadsorption was carried as described previously [40], [41], [59].

### Western blot detection and immunofluorescence localization

Western blot was performed to analyze the adult tissue distribution of grouper β-defensin. The healthy grouper tissues were homogenized in 1 ml of chilled RIPA buffer (150 mM NaCl, 50 mM Tris-HCl pH 7.2, 1% NP40, 0.1% SDS, 1% Triton X-100, 1% Deoxycholic acid, 1 mM EDTA, 1 µg/ml peupeptin, 25 µg/ml aprotinin and 1 mg/ml PMSF). Protein concentration was determined by the BCA Protein Assay Kit (Pierce, USA) according the instruction. 50 µg protein of samples from liver, kidney, spleen, fat, heart, muscle, pituitary, hypothalamus, telencephalon, cerebellum, midbrain, medulla oblongata, ovary, and testis were loaded into each lane with a ultra-low molecular weight protein size Marker (Kayon, Shanghai), electrophoresed through 16% Tricine-SDS-PAGE, then electroblotted onto PVDF membrane (Whatman, USA) using a Trans-Blot SD Semi-Dry Transfer Cell (Bio-Rad, USA). Blotting was performed according to the procedure as described previously [63], [64].

Immunofluorescence were performed to analyze grouper β-defensin celluar localization in pituitary and gonads at different development stages. The procedure was according to a previous report [19], [65].

### Grouper β-defensin promoter cloning and luciferase assay

To further analyze the regulation of grouper β-defensin expression, we cloned the its upstream region with the Universal GenomeWalker™ Kit (Clontech). As previously described [66], we isolate the promoter by using primers four pair primers (GSP1a/AP1, GSP1b/AP2, GSP2a/AP1, GSP2b/AP2). Series of PCR fragment were generated by PCR with 11 forward primers containing *SacI* and a reverse primer containing *XhoI* (Table 2). All the 5′ deletion constructs were verified by sequencing.

CO cells and EPC cells were transfected with 0.5 µg series plasmid and 0.025 µg pRL-TK as internal control, while pGL3-basic was used for negative control. After transfection for 24 h, Luciferase activities were detected with a Junior LB9509 Luminometer (Berthold, German) and normalized to the amount of Renilla luciferase activities. The results showed the representative of more than three independent experiments performed in triplicates.

### Antibacterial activity assay

*Escherichia coli* (AB91012, CCTCC), *Micrococcus luteus* (AB91100, CCTCC), *Pseudomonas aeruginosa* (AB91095, CCTCC), *Bacillus cereus* (AB93071, CCTCC) and *Staphylococcus aureus* (AB94004, CCTCC) were from China Center for Type Culture Collection (CCTCC, Wuhan). *Vibrio fluvialis*, *Vibrio anguillarum* and *Aeromonas sobria* were gifted by professor Ai-hua Li (Institute of Hydrobiology, CAS). Antibacterial activity assay was performed by microtitre broth dilution method [61], [67]. Briefly, mid-log-phase cultures of test strains were diluted to approximately 2×10^6^ CFU/ml in 2×MHB. 50 µl of these cultures were treated with 50 µl gradient concentration recombinant grouper β-defensin in 96-well microplates, while 50 µl 20 mM Tris-HCl buffer (8.0) was used as a control. The bacteria growth was determined by measuring the absorbance at 600 nm (OD_600_). Initial OD_600_ values were measured and recorded. Then these plates were incubated for 12 h at their optimal growth temperature, respectively (*S. aureus*, *P. aeruginosa* and *E. coli* at 37°C, *B. cereus* at 30°C, *V. anguillarum, A. sobria*, *M. luteus* and *V. fluvialis*, at 28°C). OD_600_ was measured and imported into Microsoft excel software and corrected by initial OD_600_ values. Survival rates were calculated as the cell density in the presence of grouper β-defensin to the cell density of control. Virtual 50% lethal dose (vLD_50_) and vLD_90_ were reported as the grouper β-defensin concentrations that resulted in survival rates of 0.5 and 0.1, respectively. Assay was performed more than three times and the statistical data were shown.

Salt sensitivity of grouper β-defensin protein was tested by adding various ions into the culture medium. 1×106 CFU/ml *E.coli* were incubated with 64 µg/ml grouper β-defensin protein containing different concentrations of salts (0–300 mM NaCl or KCl, 0–0.2 mM CaCl_2_ or MgCl_2_).

### mRNA microinjection and bacterial infection

Grouper β-defensin ORF was subcloned into the pCS2+ vector for *in vitro* transcription. After *Not I* digestion in pCS2+-grouper β-defensin, capped sense RNAs were synthesized using SP6 RNA polymerase and the SP6 Cap-Scribe (Roche, Switzerland). Following the manufacture's instructions, mRNAs were resuspended in water and injected 1 µl to an embryo at a concentration of 200 ng/µl. The expression of grouper β-defensin protein was detected by Western blot.

For bacterial infection, rare gudgeon (*Gobiocypris rarus*) embryos were manually dechorionated at 24 hpf after injected with 1 µl of the groupr β-defensin mRNA. As wild type control, the fertilized eggs were also injected with 1 µl of water. Then all the embryos were exposed to pathogen bacterium *Vibrio flurialis* (10^8^ CFU/ml) or *Micrococcus luteus* (10^8^ CFU/ml) by static immersion as described previously [68]. Embryos were maintained with fresh water after immersion for 4 h. After 12, 24, 36, 48, 60 and 72 hpi (hours post infection), the survival rates were counted. The experiments were repeated three times with 70 embryos per group.

### Transfection, virus infection and antiviral effect evaluation

CO cells and EPC cells were maintained in TC199 medium supplemented with 10% fetal bovine serum (FBS), 100 U/ml penicillin, and 100 ug/ml streptomycin at 25°C. Transfection assay was performed as described previously [44], [69]. The PCR products amplified by primers (BDF3/BDR3, Table 2) inserted into pcDNA3.1(-) vector to generate pcDNA3.1-grouper β-defensin. When they were grown to 90% confluence, EPC cells were transiently transfected with various concentration of pcDNA3.1-grouper β-defensin or empty vector pcDNA3.1(-) using the Fugene (Roche) according to the manufacturer's instruction. The expression of grouper β-defensin was detected by RT-PCR and Western blot. 24 h after transfection, the cells of each well were washed three times and treated with various concentration of RGV. After infection for 48 h, the supernatants were harvested from plates freezed and thawed for three times. For determining the titer of RGV, the supernatants were serially diluted in free-serum medium and titrated on EPC cells. Each dilution was assayed in three replicates.

All data are presented as mean ±S.E. Differences between groups were analyzed by t-test. Data were considered have significantly different when P<0.05.
